# A novel green chemistry gelation method for polyvinyl pyrrolidone (PVP) and dimethylpolysiloxane (silicone): microwave-induced in-liquid-plasma†

**DOI:** 10.1039/d1ra03007h

**Published:** 2021-07-12

**Authors:** Satoshi Horikoshi, Seiya Sawada, Nick Serpone

**Affiliations:** Department of Materials and Life Sciences, Faculty of Science and Technology, Sophia University 7-1 Kioicho, Chiyodaku Tokyo 102-8554 Japan horikosi@sophia.ac.jp; PhotoGreen Laboratory, Dipartimento di Chimica, Universita di Pavia Via Taramelli 12 Pavia 27100 Italy nick.serpone@unipv.it

## Abstract

The focus of this article rests on our discovery that a water-soluble polymer could be cross-linked to form a gel using a novel Green Chemistry gelation method: the microwave-induced in-liquid-plasma (MILP) method that requires neither a cross-linking agent nor an initiator as are required in the conventional chemical method. For instance, the water-soluble polyvinyl pyrrolidone (PVP) polymer was gelled by MILP plasma irradiation within a few minutes without using toxic cross-linking agents and initiators. As well, the hydrophobic dimethylpolysiloxane macromolecule was dispersed in aqueous media to a colloidal sol, which could then also be easily gelled under MILP irradiation conditions within a few minutes, in comparison to the conventional method that often requires several hours to days for gelation to occur in the presence of cross-linking agents and initiators. The viscosity of the MILP silicone gel was greater than a similar gel formed by the conventional method. In contrast, the viscosity of the MILP-formed PVP gel was lower than the viscosity of the PVP gel obtained from the conventional method. Gels were characterized by ^13^C-NMR spectrometry, FT-IR spectroscopy, SEM microscopy, viscosity measurements, and dynamic light scattering for particle size distributions. Plausible mechanistic stages for the two gelation occurrences have been inferred as involving the synergistic effects from microwaves, together with the sound waves (cavitation microbubbles), heat, UV and ˙OH radicals resulting from the microwave-generated in-liquid-plasma.

## Abstract 2

The discovery of a water-soluble polymer that cross-links to form a gel using a novel green gelation method: the microwave-induced in-liquid-plasma method that requires neither a cross-linking agent nor an initiator as are required in the conventional chemical method.

## Graphical abstract

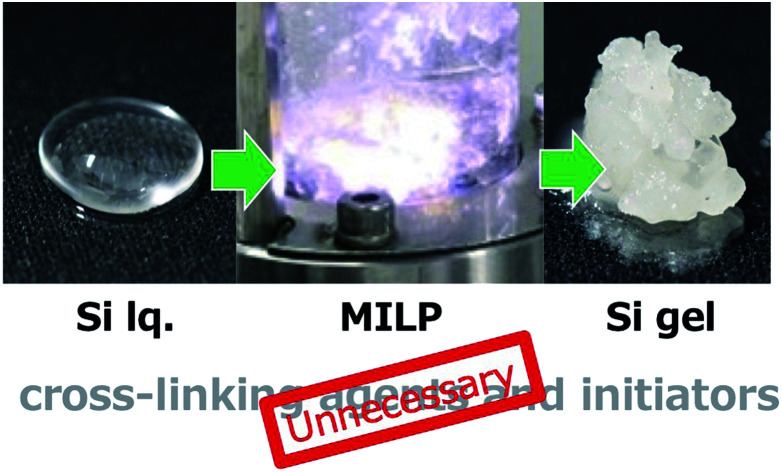

## 1. Introduction

A study by Goldston and Rutherford^1^ described a plasma as being first and foremost an ionized gas formed whenever (i) a solid is sufficiently heated so as to break the lattice and yield a liquid, which (ii) if also heated appropriately causes the atoms at the liquid surface to vaporize to a gas that in turn (iii) when also heated sufficiently the electrons are knocked off to form the plasma. Thus, plasma represents nothing more than a group of positively and negatively charged particles, while remaining electrically neutral as a whole; that is, plasma can be viewed as a small part of a neutral gas of ionized atoms or molecules. The earliest report of artificially generated plasma was achieved in Crookes' tube in 1879.^2^ Since then, basic and applied researches on plasma have been conducted worldwide, and it is now recognized that when thinking plasma it would be in the gaseous form. Nearly a century later (1987), a completely new method was proposed by Clements *et al.*^3^ to generate plasma; these authors reported that plasma could be generated (substance changing into a plasma) by applying an AC high-voltage pulse discharge into water, for example, that led to the development of what is now referred to as submerged plasma or as in-liquid-plasma. This leading study further suggested that the plasma generated in a liquid phase is significantly different from the plasma generated in a gaseous phase. In 2003, Nomura and Toyota^4^ irradiated a metal electrode in a solution with microwaves with the heat vaporizing the liquid around the metal electrode that resulted in the formation of bubbles near the electrode and in the generation of plasma within the bubbles. Conditions for generating plasma in a liquid need not, however, be an electrolyte solution as plasma can also be generated under atmospheric pressure.^4^ In fact, submerged plasma can be generated without depending on conductivity, temperature, and atmospheric pressure, so that various applications have been developed for the use of plasma. For instance, a microwave-induced submerged plasma device has been used (a) to synthesize metal nanoparticles,^5,6^ (b) to dope photocatalysts with some foreign atoms,^7^ and (c) for the treatment of waters,^5,8^ among others.

However, to the extent that such a device concentrates microwaves on the tip of a microwave antenna electrode to generate the submerged plasma, the tip of the antenna tends to be damaged (melt) by the heat and by the cavitation of the in-liquid-plasma, so that plasma could not be generated continuously. Nonetheless, in our previous study,^9^ we succeeded in generating microwave-induced in-liquid-plasma (MILP) continuously under microwave pulsed oscillation conditions that precluded the accumulation of heat at the tip of the antenna electrode and thus avoid damaging the antenna. Accordingly, we were able to degrade fairly rapidly such pollutants as the persistent perfluorooctanoic acid,^10^ dye-contaminated wastewaters,^9^ dioxane,^11^ and the flame retardant tetrabromobisphenol-A in alkaline aqueous media.^12^

**Fig. 1:**
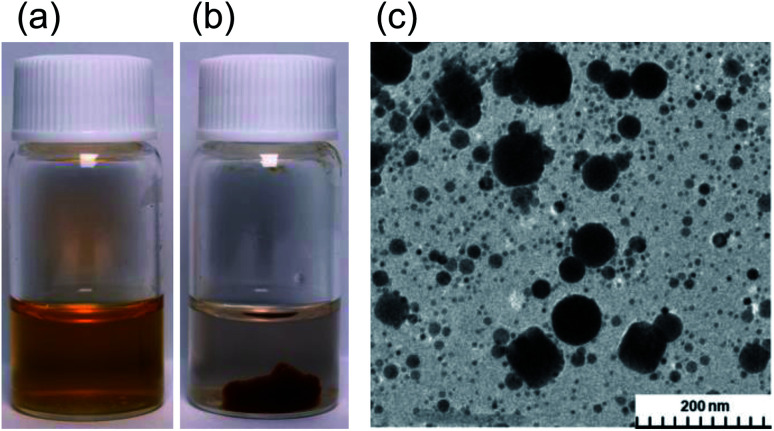
Synthesized Ag nanoparticles in the presence of polyvinylpyrrolidone (PVP) in aqueous media using (a) the traditional microwave heating method and (b) by the microwave-induced in-liquid-plasma (MILP) method. (c) TEM image of Ag nanoparticles synthesized by the MILP method.

The present study started from a completely unexpected discovery during an earlier study^13^ in which our strategy was to synthesize silver nanoparticles using MILP to irradiate a 50 mL aqueous solution of silver nitrate for 5 min in the presence of polyvinyl pyrrolidone (PVP; a protective and reducing agent). At first, the synthesis of Ag nanoparticles carried out with simple microwave heating, rather than MILP albeit under the same conditions, yielded a clear light brown aqueous solution due to plasmon absorption of the Ag nanoparticles (Fig. 1a). Allowing the Ag nanoparticulate sol containing the PVP (Fig. 1a) to stand for 3 days did not lead to gelation of the PVP polymer. On the other hand, when the silver nitrate/PVP solution was irradiated by the MILP method, formation of silver nanoparticles was visually observed from the color change of the gel-like substrate, although the particulates were embedded in a gel-like (jelly-like) mass (Fig. 1b). Vacuum freeze-drying the gel-like mass (Fig. 1b) and observations from transmission electron microscopy (TEM) revealed highly irregular Ag nanoparticles contained within the gel (Fig. 1c). These observations confirmed the synthesis of metal nanoparticles by the submerged plasma (MILP) in which the PVP acted as both a protective agent and/or as a reducing agent.

In general, gel formation by the conventional method necessitates the presence of either a cross-linking agent and/or an initiator in some organic solvent, some of which are highly toxic (Table S1 of ESI†) and so require additional chemicals and stages for the purification of the gels formed. Accordingly, this process does not conform to the first five of Green Chemistry's twelve principles.^14^ In contrast, the MILP method necessitates neither the use nor the need to purify these reagents, and thus incurs no additional unnecessary costs.

The principal objective of the present study was to elucidate the characteristics of this novel phenomenon discovered by sheer serendipity and involved a cross-linking reaction (gelation reaction) of macromolecules by the MILP method. In particular, the study (i) examined the structure and physical properties of the PVP gels, (ii) optimizing the conditions for the gel synthesis, and (iii) exploring further possibilities as for example the formation of silicone gels.

## 2. Experimental setup

### 2.1 The MILP device

**Fig. 2:**
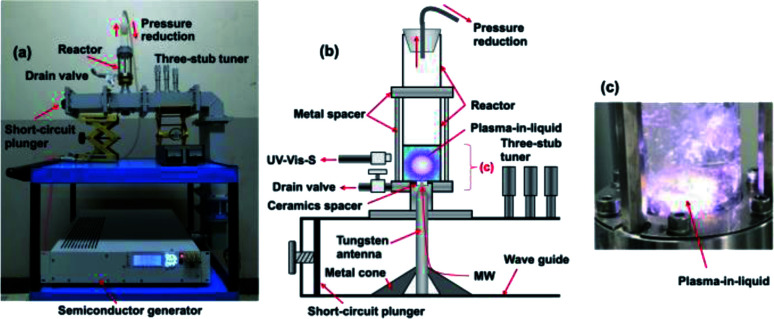
(a) Photograph and (b) schematic illustration of the in-liquid plasma device; (c) photograph of the generated in-liquid plasma in the quartz reactor.

Fig. 2a and b illustrate, respectively, a photograph and a schematic of the microwave-induced in-liquid-plasma (MILP) device, while Fig. 2c displays the microwave-generated in-liquid-plasma in an aqueous PVP solution. The microwave device was constructed of an Ampreon M2A-R semiconductor generator that emitted pulsed microwaves (maximal power, 1500 W; microwave frequency, 2.4500000 GHz), an isolator (air cooling device), a power monitor, a three-stub tuner, and a short-circuit plunger. To prevent deterioration of the electrodes from the microwave-generated plasma heat, pulsed irradiation was performed under otherwise identical conditions to those described earlier.^9^ Optimal conditions were irradiation with 150 W microwaves (the pulse amplitude); pulse period was 16.7 ms and the pulse width was 13.36 ms.

The liquid medium was continuously irradiated with microwaves through the tungsten antenna (diameter, 1.0 cm; length, 20.0 cm). Metal cones in the waveguide focused the microwaves onto the tungsten antenna tip, through which the microwaves irradiated the aqueous solution and generated the in-liquid-plasma (Fig. 2b). The tungsten antenna was isolated from both the reactor and the waveguide using a ceramic spacer, so that the microwaves irradiated only the liquid. The cylindrically shaped reactor consisted of a quartz vessel (diameter, 32.0 mm; length, 160.0 mm) fabricated such that the pressure could be reduced with a water aspirator through the top of the reactor. Note that the size of the quartz reactor was determined from the distribution of the electromagnetic waves.^9^ The irradiating microwaves were adjusted by the three-stub tuner such that there were no reflected waves.

Results from the gel synthesis under MILP irradiation show that the gel was not generated continuously at constant plasma density, and although microwave irradiation used constant microwave pulses, the MILP was generated with irregular pulses with the intensity of the plasma depending on the temperature, the state, and the bubble conditions within the solution. In addition, gel irradiation time was not constant for each sample. Consequently, the microwave irradiation time to generate MILP was taken as the time from turning the microwaves ON to the time of turning them OFF. Each experiment was carried out three times to establish there were no significant differences between the three runs.

### 2.2 Synthesis of the PVP gel

Polyvinyl pyrrolidone (PVP; Tokyo Kasei Co. Ltd) with average molecular weight of 40 000 was used in the synthesis of the PVP gel by dissolving 5.0 g of PVP in 50.0 mL of ion-exchanged water in the quartz reactor. The solution was then irradiated with 150 W pulsed microwaves to generate the submerged plasma (MILP) that in turn led to the formation of the PVP gel, which was collected from the drain valve (Fig. 2b) along with the aqueous solution.

The physical and chemical characteristics of the MILP-synthesized PVP gels were then compared with similar PVP gels synthesized by the conventional chemical synthesis method in the presence of both the *N*,*N*′-methylenebisamine (0.5 g; Fujifilm Wako Chemicals Ltd) cross-linker and the potassium persulfate initiator (K_2_S_2_O_8_; 0.1 g; Fujifilm Wako Chemicals Ltd) in an aqueous solution of PVP (5.0 g; 50.0 mL); the solution was subsequently heated in a water bath at 80 °C for 5 min as recommended by the reagent manufacturers. We also produced a PVP gel by MILP irradiation for 1 min a 50.0 mL aqueous solution of PVP (5.0 g) containing the cross-linking agent but without the initiator.

To simplify the elucidation of the mechanism of PVP gelation, we used the monomeric *N*-methylpyrrolidone (NMP: Tokyo Kasei Co., Ltd) as our model compound of PVP by dissolving 5.0 g of NMP in 50.0 mL of ion-exchanged water followed by MILP irradiation for 5 min.

### 2.3 Synthesis of the silicone gel

The synthesis of the dimethylpolysiloxane precursor was carried out by a known method^15,16^ that involves the monomeric dichlorodimethylsilane as the starting material (reaction (1)). The process involved dissolving 5.0 g of this silane in 50 mL of ion-exchanged water followed by stirring at 25 °C for 1 h and then at 60 °C for 5 h in a water bath.1
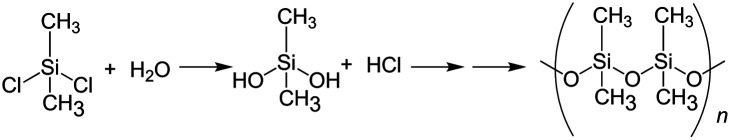


For the synthesis of the silicone gel by the MILP method, 5.0 g of the dimethylpolysiloxane from reaction (1) was placed in ion-exchanged water and subsequently irradiated with the MILP submerged plasma for 30 s with the 150 W pulsed microwaves. To compare the physical and chemical properties of the resulting silicone gel with the one from the conventional chemical method, we added 5.0 g of the dimethylpolysiloxane, 10.0 g of tetraethyl orthosilicate (TEOS), 0.44 g of hydrochloric acid, 1.30 g of water, 1.92 g of ethanol, and 3.46 g of tetrahydrofuran (THF) to the quartz reactor. Subsequent mixing and allowing the mixture to stand for 3 days yielded the silicone gel.^15^ Note that in general gelation times by the conventional method can be from a few hours to a few days depending on the temperature and the solution pH.^17^

### 2.4 Analytical and characterization methods

Particle size distributions were obtained by dynamic light scattering using an Otsuka Electronics Co., Ltd, DLS instrument (model ELSZ-2000 ZS), while the elemental analysis was carried out with a fully automated elemental analyzer using the frontal chromatographic method (Perkin Elmer Co., Ltd, PE2400 Series II CHNS/O). Fourier transform infrared spectra (FT-IR), Raman spectra and UV/Vis absorption spectra were obtained, respectively, on a JASCO Co. FT/IR-4600 spectrophotometer with an attenuated total reflection (JASCO Co. ATR PRO ONE), a JASCO Co. NRS-4500 Raman spectrometer, and a JASCO Corporation V-760 spectrophotometer. Carbon-13 and proton nuclear magnetic resonance (NMR) spectrometry were performed with a JEOL Ltd Lambda 500 MHz spectrometer; analysis of the NMR data was carried out on the JEOL Ltd ALICE system. X-ray photoelectron spectroscopy (XPS) and scanning electron microscopy (SEM) used, respectively, an ULVAC-Phi ESCA 5800ci spectrometer and a Hitachi High Technologies Co., Ltd, SEM, SU8000 electron microscope. The viscosity of the gel was assessed with a Toki Sangyo Co., Ltd R215 viscometer.

^13^C-NMR and ^1^H-NMR analyses were done on dimethylpoly-siloxane and silicone gel samples with chemically cleaved Si–O bonds in the silicone gel. Experimentally, each 0.5 g of dimethylpolysiloxane or silicone gel (after MILP irradiation) were added to a mixed solution of 2.27 g methyl orthoformate, 0.699 g methanol and 0.002 g sulfuric acid and then stirred at room temperature for 3 days. This operation led to the separation of the Si–O bond portion of the silicone gel.

Molecular orbital (MO) calculations of frontier electron densities of each element were carried out on our model system *N*-methylpyrrolidone at the single determinant (Hartree–Fock) level to assess the potential positions of attack by the generated radicals during gel formation of PVP. The optimal conformation of minimum energy was obtained semi-empirically at the AM1 level. All semi-empirical calculations were performed with the MOPAC 97 and the CAChe package implemented on a Windows computer system.^18,19^

## 3. Results and discussion

**Fig. 3:**
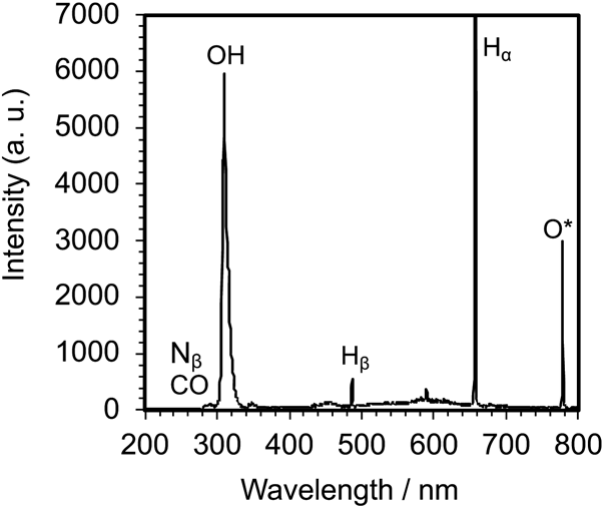
Spectrum of the light emitted from the generated MILP in an aqueous PVP solution.

Irradiation of an aqueous PVP solution with 150 W pulsed microwaves led to the emission of a whitish-blue light (Fig. 2c) that confirmed the generation of the microwave-induced in-liquid-plasma (MILP). Recording this emitted UV-Vis light through an optical fiber with the UV/Vis spectrophotometer (Fig. 3) revealed a spectrum that consisted of three relatively intense emission lines attributable to ˙OH radicals (316 nm), H_α_ hydrogen (656 nm) and O* oxygen (778 nm).^20–22^ Peaks belonging to N_β_ nitrogen (289 nm), CO (298 nm) and H_β_ hydrogen (484 nm) were also detected, albeit less intense. Note that the plasma was generated within the microbubbles formed near the antenna tip.

### 3.1 Establishing and optimizing the formation of the PVP gel

**Fig. 4:**
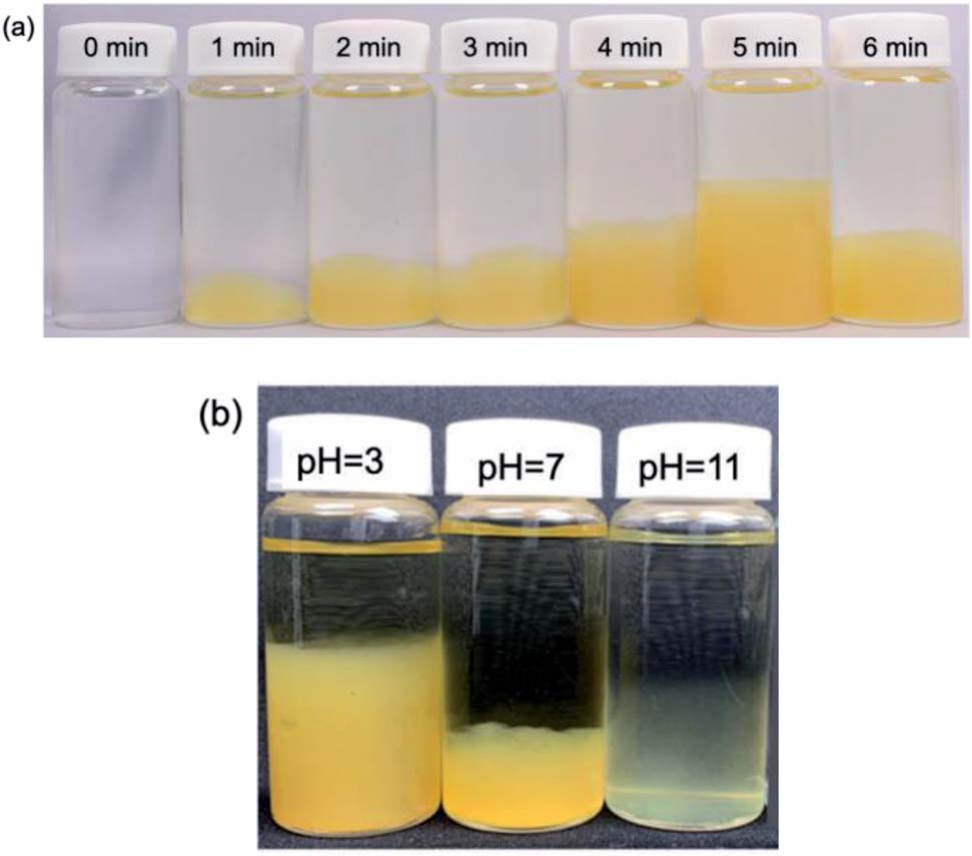
(a) Photographs of a PVP gel taken after MILP irradiation times of 0, 1, 2, 3, 4, 5 and 6 min and standing for 24 h. (b) Photograph of the PVP gel after 3 min of MILP irradiation at different pHs (pH = 3, 7 and 11).

Irradiation of an aqueous AgNO_3_-free PVP solution with the MILP method for 5 min turned a transparent colorless solution (*t* = 0 min; Fig. 4a) into a fluffy yellow gel consisting of fine solid particles. This clearly established that formation of the PVP gel required neither AgNO_3_ nor Ag nanoparticles, but rather was caused solely by the submerged plasma. Nonetheless, as the initial colorless PVP solution turned yellow with increasing MILP irradiation time, we tentatively deduced that the PVP was decomposed by the plasma and that some intermediate species underwent some sort of cross-linking stage to form the gel.

To establish that the PVP solution irradiated with the MILP had indeed formed a gel, the yellow fluffy substance was collected, filtered, and dried; portions of the dried PVP gel were subsequently immersed in 9 different solvents (water, methanol, ethanol, acetone, hexane, chloroform, dimethylformamide, dimethyl-acetamide, tetrahydrofuran) and allowed to stand for 24 h to determine their solubility and the presence or absence of swelling (Fig. S1; see ESI†). Results showed that the PVP gel particulates were completely insoluble in all solvents, and there was no swelling in these solvents. Accordingly, the yellow PVP sample was undeniably already in a gel state.

Viscosity is also an important property of gels. Accordingly, the viscosity of the PVP gel produced by the MILP method was measured at an angular velocity of 10 rad s^−1^ that gave 5.01 Pa s, while the viscosity of the PVP gel formed by the conventional method in the presence of a cross-linking agent and an initiator was 89.8 Pa s. Evidently, the PVP gel formed by the submerged plasma with neither a cross-linking agent nor an initiator present was less viscous and had a degree of cross-linking that was less than 6% of the gel formed by the conventional method. On the other hand, a PVP aqueous solution containing a cross-linking agent, which was then plasma-irradiated for only 1 min led to the formation of a PVP gel whose viscosity increased significantly (96.2 Pa s). This demonstrates that cross-linking can be enhanced by the MILP method with no need for an initiator or thermal energy. Nonetheless, the viscosity of the PVP gel was further enhanced, albeit slightly, in the presence of the initiator and with thermal energy.

The next phase of our study involved determining, albeit visually, the optimal irradiation times to produce a PVP gel with MILP irradiation. Fig. 4a displays a series of photographs of a PVP aqueous solution taken 1 day after the solution was irradiated with 150 W pulsed microwaves for 1, 2, 3, 4, 5 and 6 min. The photographs show the quantity of PVP gel formed increased with MILP irradiation time, although the amount of PVP gel produced decreased when the irradiation time exceeded 5 min. We suspected that the ˙OH radicals (redox potential of +2.31 V at pH = 7)^23^ generated by submerged plasma irradiation may be responsible for some of the PVP gel to decompose. Nevertheless, the data in Fig. 4a infer that the threshold time for the synthesis rate was faster than the rate of decomposition for times ≤5 min. Note that this time threshold may change with increasing intensity of the microwave-induced in-liquid-plasma.

The tendency for the PVP aqueous solution to gel under MILP irradiation was also examined at different pHs (pH = 3, 7 and 11) adjusted with hydrochloric acid or sodium hydroxide. Each PVP solution was MILP-irradiated for 3 min, and then allowed to stand for 24 h before taking the photographs displayed in Fig. 4b. Unmistakably, the amount of PVP gel formed at pH = 3 was significantly greater than at pH = 7, and more so than in alkaline media at pH = 11. Earlier we discovered that the number of ˙OH radicals formed under MILP conditions in aqueous media increased with increase in pH,^12^ which infers that the rate of decomposition of the PVP and/or the PVP gel exceeds the rate of formation of the gel at the higher pHs.

The two results illustrated in Fig. 4 further infer that the ˙OH radicals are somehow also critically involved in the gelation mechanism of PVP. Accordingly, to the extent that highly active radicals may also be implicated in driving the gelation process, it is imperative to adjust the balance between formation and decomposition of the gel by adjusting the pH.

### 3.2 Elucidation of the mechanism of PVP gel formation

**Fig. 5:**
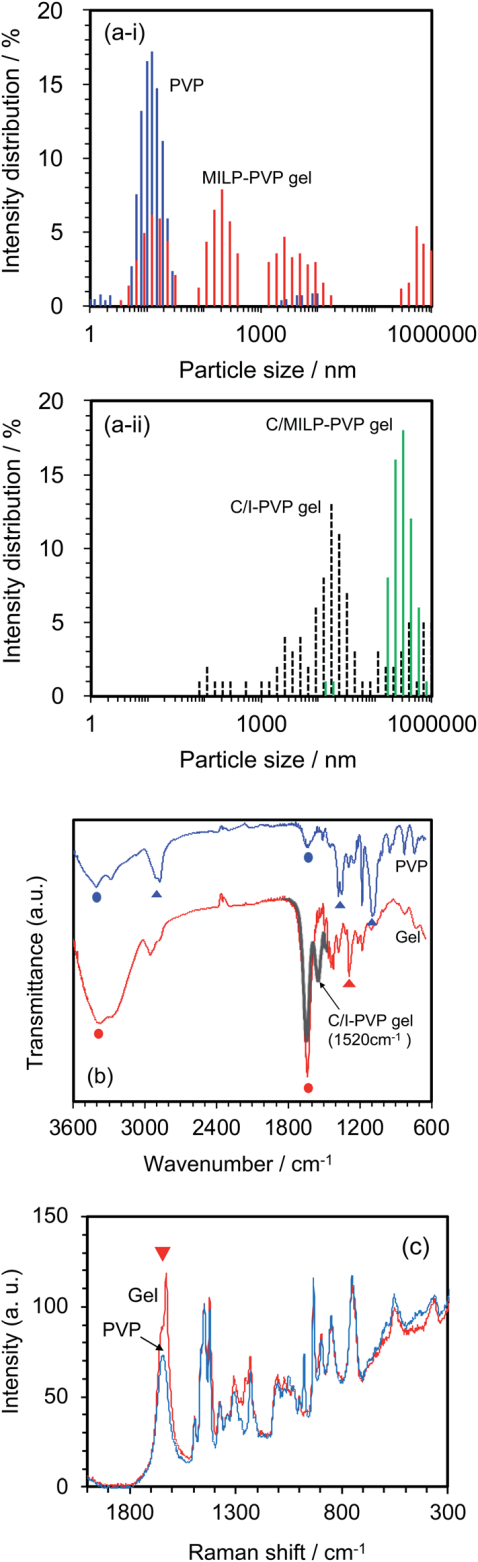
Comparison of PVP before MILP irradiation (blue) with PVP gel after MILP irradiation (red): (a-i) particle size distributions; (a-ii) particle size distributions of PVP from conventional gel synthesis method with cross-linking agent and an initiator (C/I-PVP gel: black dotted line) and MILP gel synthesis method with cross-linking agent (C/MILP-PVP gel: green line); (b) FT-IR spectra, (c) Raman spectra. Circles in (b) refer to vibrational modes in both the PVP and the PVP gel, while the triangles denote loss of the vibrational modes in PVP following PVP gelation. The black line of the partial spectrum in (b) refers to the conventional gel synthesis method with the cross-linking agent and an initiator.

Changes in the size distributions of the PVP macromolecule after a 3 min MILP irradiation period were confirmed by dynamic light scattering of both the PVP polymer and the resulting gel. The initial PVP in aqueous media displayed a somewhat narrow particle size distribution centered around 10 nm (Fig. 5a-i); however, irradiation of the PVP with submerged plasma for 3 min increased the molecular size distributions by factors of 10, 100, and 100 000 (*i.e.*, sizes centered around 100 nm, 1000 nm, and 1 000 000 nm). On the other hand, with the conventional gel synthesis method with cross-linking agent and an initiator (C/I-PVP gelz), the gel size is widely distributed from 1000 to 1 000 000 nm (Fig. 5a-ii). However, the size range was narrower when the synthesis of the PVP-gel with the cross-linking agent alone was carried out under MILP irradiation without the initiator.

Structural changes that occurred subsequent to MILP irradiation of a PVP solution to produce the PVP gel were investigated by FT-IR spectroscopy. Fig. 5b displays the FT-IR spectrum of PVP before MILP irradiation, which shows vibrational modes at 3410 cm^−1^ attributable to the –OH group, and at 2905 cm^−1^ (alkyl chain CH_2_ group), 1631 cm^−1^ (amide I, C=O), 1378 cm^−1^ (C–H), and 1099 cm^−1^ (C–C and –CH_2_ groups).^24^ By comparison, the FT-IR spectrum of the PVP gel shows significantly increased peak intensities for the –OH group near 3410 cm^−1^ and the C=O peak at 1631 cm^−1^, likely the result of additional OH groups on the PVP structure. On the other hand, comparison of the FT-IR spectrum of the PVP gel formed by the MILP method and by the conventional gel synthesis method in the presence of a cross-linking agent and an initiator reveals a new peak at 1520 cm^−1^ in the gel from the conventional method (Con-Gel; black line that overlaps the red line in Fig. 5b.), which is attributed to the amide II (N–H bending, and C–N stretching).^25^ The peak at 1520 cm^−1^ derived from this new amido is attributed to the *N*,*N*′-methylenebisamine cross-linker. However, as this peak is not seen in the PVP gel by the MILP method suggests that PVP undergoes decomposition by the MILP and the structure R–CO–NH–R is not generated. The Raman spectra of Fig. 5c show significantly enhanced Raman peaks around 1605 cm^−1^ that are assigned to the C=O stretching vibration.^26^

The constituent atom mass ratios of PVP and PVP gel (MILP method) were measured by an elemental analysis; they are reported in Table 1 for nitrogen (N), carbon (C), hydrogen (H) and oxygen (O). Cross-linking from PVP to the PVP gel caused the nitrogen to decrease from 12.6% to 9.6%, and the carbon to decrease from 64.8% to 49.1%; the hydrogen ratio decreased from 8.2% to 7.0%, while the oxygen increased significantly from 14.4% to 34.3%. The increase in oxygen is likely due to an increase in OH groups in the process of PVP gelling. This result infers that gelation proceeds by a plasma-induced oxidation reaction.

**Table 1:** Constituent atom mass ratios (in %) for nitrogen (N), carbon (C), hydrogen (H), and oxygen (O) in pristine PVP and in a PVP gel

Sample	Mass percent/%			
	Nitrogen (N)	Carbon (C)	Hydrogen (H)	Oxygen (O)
PVP (before MILP)	12.6	64.8	8.2	14.4
PVP gel (after MILP)	9.6	49.1	7.0	34.3

These results deduce that MILP irradiation of the aqueous medium led to the homolytic dissociation of water yielding ˙OH radicals (UV wavelength, 316 nm; Fig. 3), which could participate (among other factors) in the gelation process by attacking the five-membered ring group in PVP molecules and cause the cross-linking process to proceed. To support this hypothesis and in order to predict the position of ˙OH radical attack on PVP, we used *N*-methyl-2-pyrrolidone (NMP) as a model monomer for PVP to facilitate the calculations of the initial ˙OH radicals attack position(s) from the calculated electron densities reported in Table 2. The atoms with the highest electron density in the NMP structure (Table 2a) were the C=O carbon atom C(2) and the ring N(7) atom. As a result, we expect the initial ˙OH radical attack to be focused on the C(2)–N(7) bond vicinity that would ultimately lead to the breakup of the heteroatom ring at that position (see below).

**Table 2:** Frontier electron density calculations on *N*-methyl-2-pyrrolidone (NMP): (a) chemical structure of NMP and definition of the atom; (b) image displaying large and small frontier electron densities

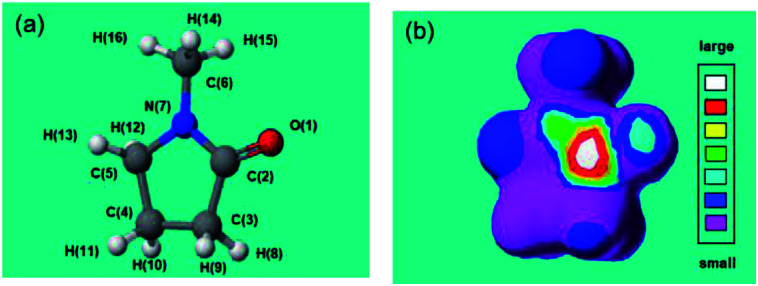			
Atom	Frontier electron density	Atom	Frontier electron density
O(1)	0.301	H(9)	0.028
**C(2)**	**0.627**	H(10)	0.008
C(3)	0.016	H(11)	0.008
C(4)	0.011	H(12)	0.065
C(5)	0.066	H(13)	0.061
C(6)	0.057	H(14)	0.065
**N(7)**	**0.606**	H(15)	0.051
H(8)	0.028	H(16)	0.001

**Fig. 6:**
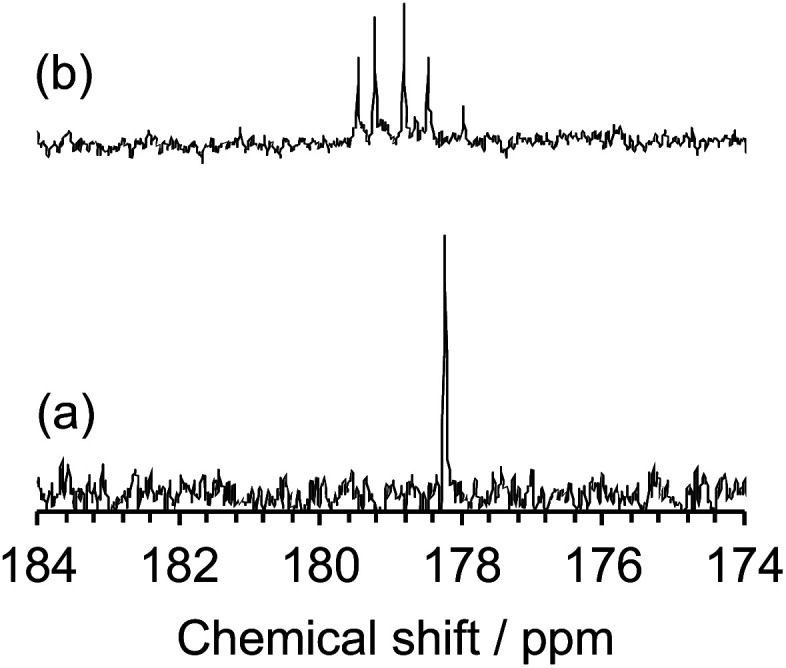
^13^C-NMR spectra from 174 to 184 ppm of (a) NMP before MILP irradiation and (b) NMP after MILP irradiation.

The inferred breakup of the five-membered ring (ring opening) subsequent to MILP irradiation of an aqueous NMP solution was examined by UV/Vis absorption spectroscopy. The UV absorption band of the five-membered ring at 283 nm saw its intensity reduced by 69% in the first 2 min to disappear completely in about 5 min. Changes in the chemical structure of the NMP before and after MILP irradiation was also confirmed by ^13^C-NMR spectrometry. The peak at 178.2 ppm attributed to the C(2)–N(7) carbon in NMP before MILP irradiation (Fig. 6a) disappeared after 5 min of MILP irradiation. In exchange, new peaks occurred at 178.0, 178.5, 178.8, 179.2, and 179.5 ppm (Fig. 6b; note that assigning these new peaks was outside the scope of the present study).

**Fig. 7:**
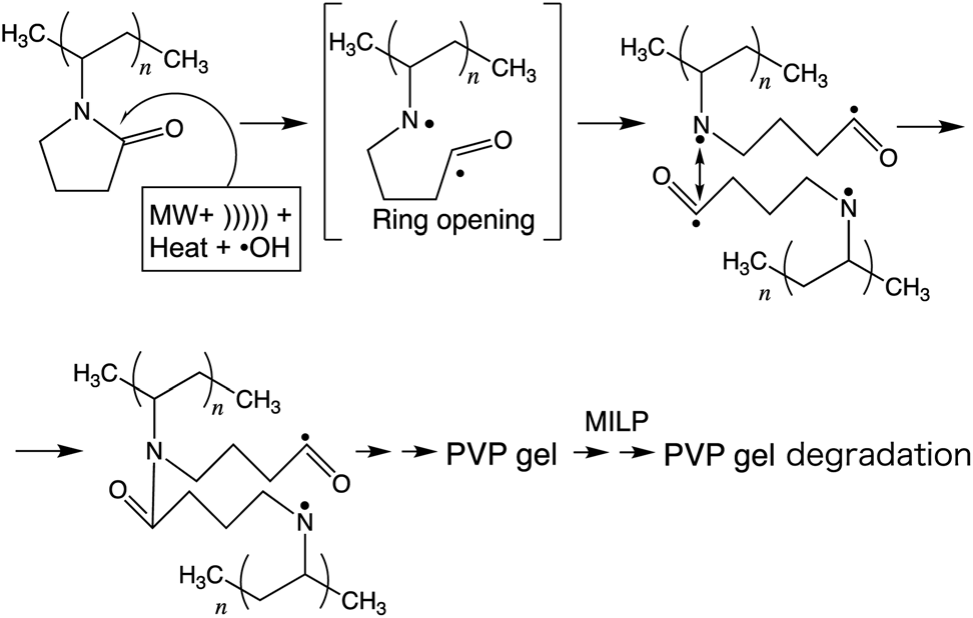
Inferred mechanistic stages into the synthesis of the PVP gel by the MILP method.

Thus, based on the above experimental results and the computed frontier electron densities of Table 2, it is not unreasonable to infer a mechanistic course for the gelation of PVP in aqueous media subsequent to MILP irradiation (see scheme in Fig. 7). As stipulated by Goldston and Rutherford,^1^ the first stage involves the generation of the in-liquid-plasma by the microwave irradiation of water occurring through loss of electrons {H_2_O + MW → MILP(H^+^ + ˙OH) + e^−^*} to yield ˙OH radicals (among others) – see the UV emitted light at 316 nm in Fig. 3 – which then could attack the five-membered heteroatom ring of PVP in the vicinity of the keto group, ultimately causing the ring to open and produce radical intermediate species. In keeping with the study of Fechine and coworkers,^27^ who reported diradical intermediates from the photo-induced transformation of PVP under UV light, it is not unlikely that under our experimental conditions (microwave radiation, in-liquid-plasma, sound waves cavitation microbubbles, and heat) similar radical species may have formed. Subsequent linking of the two radicals from two PVP macromolecules generates a new amide bond. In due course, repeated such events lead to the gelation of PVP.

To the extent that the MILP generates ˙OH radicals, sound waves, UV, and heat,^5^ it is reasonable to infer that the rate-determining factor of gel formation involves radical reactions. To ascertain such an inference, an aqueous solution of PVP was also subjected to ultrasonic waves from a homogenizer, as well as being exposed to UV radiation using a low-pressure mercury lamp for 30 min. Results from these two experiments showed no occurrence of gel formation. Accordingly, gelation must have resulted from some radical reactions following the synergistic effects of microwaves, sound waves, UV, heat, and ˙OH radicals. No doubt that under our experimental conditions, gelation of PVP occurred *via* a very complex route, details of which remain elusive within the present study, so that the stages illustrated in Fig. 7 must be considered as a work in progress.

### 3.3 Gelation of dimethylpolysiloxane (silicone)

**Fig. 8:**
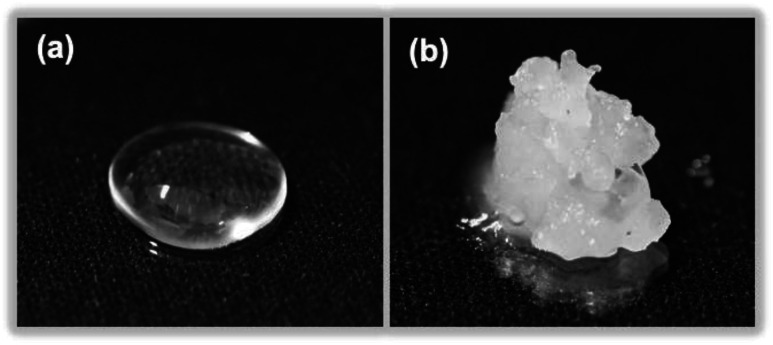
Photographs of (a) dimethylpolysiloxane (silicone) and (b) a silicone gel produced after a 5 min MILP irradiation period.

Dimethylpolysiloxane (also referred to as silicone) is insoluble in ion-exchanged water and thus yields a transparent colloidal sol (Fig. 8a), which when exposed to MILP irradiation for 5 min is transformed into a white spongy-like hydrogel (Fig. 8b). Accordingly, the MILP method can also convert water-insoluble macromolecules into gels. Regardless, the silicone gels were then added to 9 solvents (water, methanol, ethanol, acetone, hexane, chloroform, dimethylformamide, dimethylacetamide, and tetra-hydrofuran) to examine their solubility and swelling (Fig. S2 of ESI†). Although the dimethylpolysiloxane was soluble in hexane, the corresponding silicone gel was insoluble in this and other solvents, thereby confirming gel formation.

**Fig. 9:**
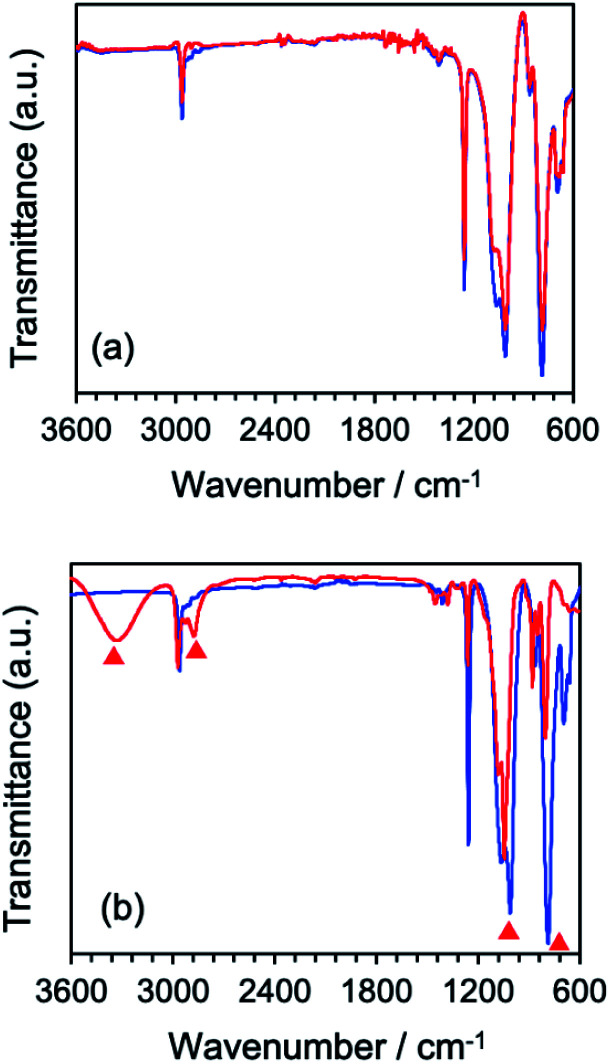
(a) Comparison of FT-IR spectra of silicone (blue) and silicone gel (red) synthesized by the MILP method, (b) FT-IR spectra of silicone (blue) and silicone gel (red) synthesized by the conventional chemical method. Red triangles in (b) refer to the appearance of new peaks or changes in peak intensities in the silicone gel *vis-à-vis* the dimethylpolysiloxane (silicone).

The difference of the chemical structure of dimethylpolysiloxane *versus* the silicone gel formed from the former after a 5 min MILP irradiation period was investigated by FT-IR spectroscopy (Fig. 9a). Comparing the FT-IR spectra of dimethylpolysiloxane against the silicone gel revealed main peaks occurring at 780 cm^−1^ (Si–C bond), 1000 cm^−1^ (Si–O bond), 1260 cm^−1^ (Si–CH_3_ bond), and 2960 cm^−1^ (CH_*x*_);^28^ no structural differences were evident. On the other hand, comparison of the FT-IR spectrum of the silicone gel synthesized by conventional means in the presence of the cross-linking agent and the initiator with the FT-IR spectrum of dimethyl-polysiloxane (Fig. 9b) revealed new peaks at 3318 cm^−1^ (O–H bond) and at 2875 cm^−1^ (C–H bond) for the silicone gel,^29^ while the peaks below 1010 cm^−1^ (Si–O) became less intense as the silicone gelled.

The results of the elemental analysis of the synthesized silicone gel are summarized in Table 3. Data for the silicone gel from the MILP method revealed that carbon (C) and oxygen (O) increased by 5.5% and 2.9%, respectively, and that silicon (Si) decreased by 8.5% compared to the silicone gel produced from the conventional method. A possible reason for the relative increase of C is the likely involvement of ˙OH radicals generated during the formation of the in-liquid-plasma that attack the methyl group of the silicone, causing a cross-linking reaction with loss of a H atom and formation of a C–C bond. The increase in O is due to substituting a H on a methyl group by an OH function following attack of silicone by the ˙OH radicals at the methyl groups. The relative decrease in silicon (Si) is mostly due to an increase in C and O *vis-à-vis* silicon that remains constant throughout the in-liquid-plasma irradiation period.

**Table 3:** Constituent atom mass percent for carbon (C), oxygen (O) and silicon (Si) in the silicone gel formed by the MILP synthesis method and by the conventional chemical synthesis method

Silicone-gel	Mass percent/%		
	Carbon (C)	Oxygen (O)	Silicon (Si)
Dimethylpolysiloxane	54.2	16.7	29.1
MILP method	36.9	50.1	12.9
Conventional chemical synthesis	31.4	47.2	21.4

**Fig. 10:**
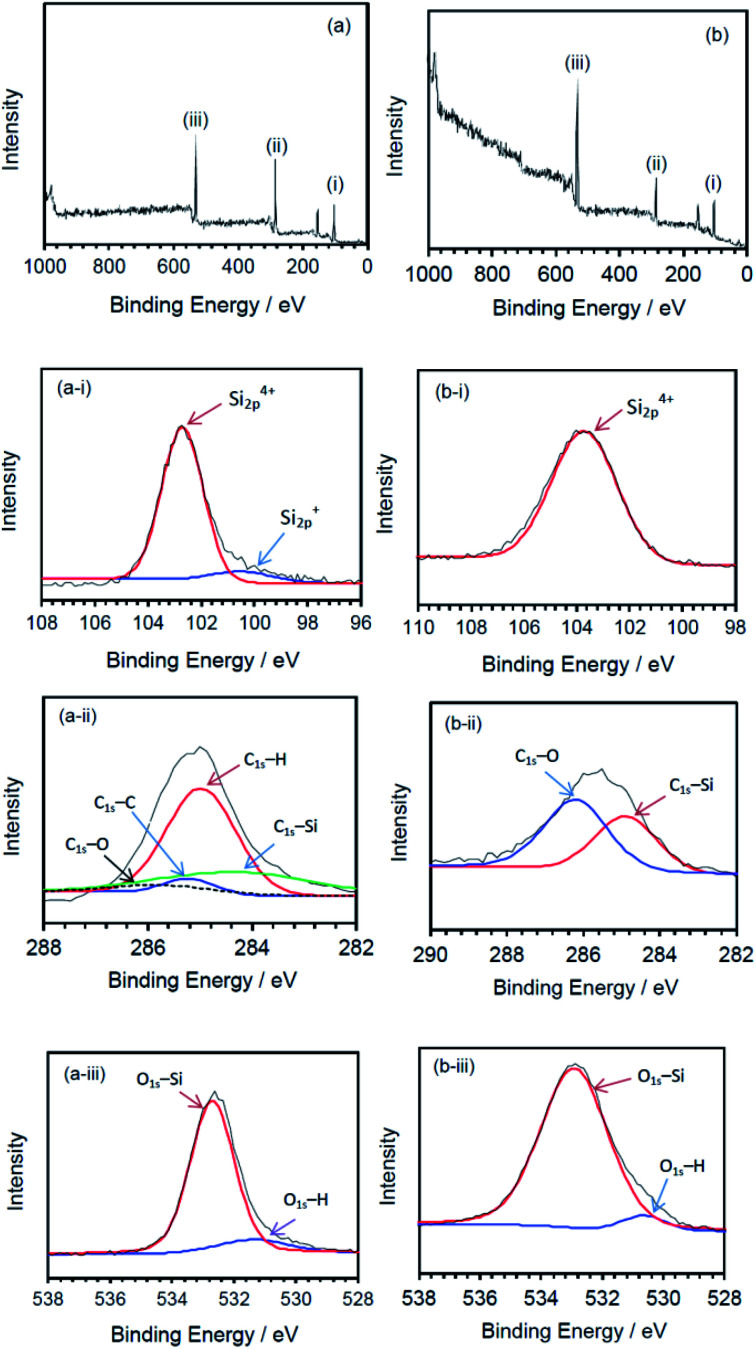
XPS spectra of silicone gels synthesized by (a) the MILP method and (b) by the conventional method; deconvoluted spectra for the (i) Si_2p_ peaks, (ii) C_1s_ peaks, and (iii) O_1s_ peaks.

The nature of the surface of the silicone gels synthesized by the MILP method and by the conventional chemical synthesis method was examined by XPS spectroscopy (Fig. 10). The principal peaks of the MILP silicone gel occurred at 103 eV (Si_2p_), 155 eV (Si_2s_), 285 eV (C_1s_) and 533 eV (O_1s_) – see Fig. 10a. In comparison, the XPS peaks of the conventional silicone gel were observed at 104 eV (Si_2p_), 155 eV (Si_2s_), 286 eV (C_1s_) and 533 eV (O_1s_) (Fig. 10b), in accord with those reported by Aghaei and Eshaghi.^30^

The waveform of each XPS spectrum was deconvoluted into Gaussian peaks using the software of the XPS device, which was also used to calculate the energies for each peak corresponding to a given constituent functional group. The MILP silicone gel (Fig. 10a-i) showed the peak of Si_2p_ to consist of a Gaussian peak for Si^4+^ (Si–O_4_) at 102.6 eV and a Gaussian peak for Si^+^ (Si_3_–Si–O) at 100.6 eV.^31,32^ There was no corresponding peak for Si^+^ (Si_3_–Si–O) in the XPS spectrum of the conventional silicone gel (see Fig. 10b-ii). Fig. 10a-ii shows the components of the C_1s_ deconvoluted peak for the MILP silicone gel that includes peaks at 284.1 eV (C–Si), 285.0 eV (C–H), 285.3 eV (C–C) and 286.2 eV (C=O),^33,34^ while Fig. 10b-ii displays peaks at 284.8 eV (C–Si) and 286.1 eV (C–O) for the conventional silicone gel.^33^ Thus, the chemical structure of the MILP silicone gel also includes C–C bonds. Fig. 10a-iii and b-iii show, respectively, the peak components of the O_1s_ peak occurring at 532.5 eV (O–Si) and at 531.3 eV (O–H) for the MILP silicone gel;^35,36^ for the conventional silicone gel the corresponding peaks occurred at 533 eV and 530.5 eV.^34,35,37^

**Fig. 11:**
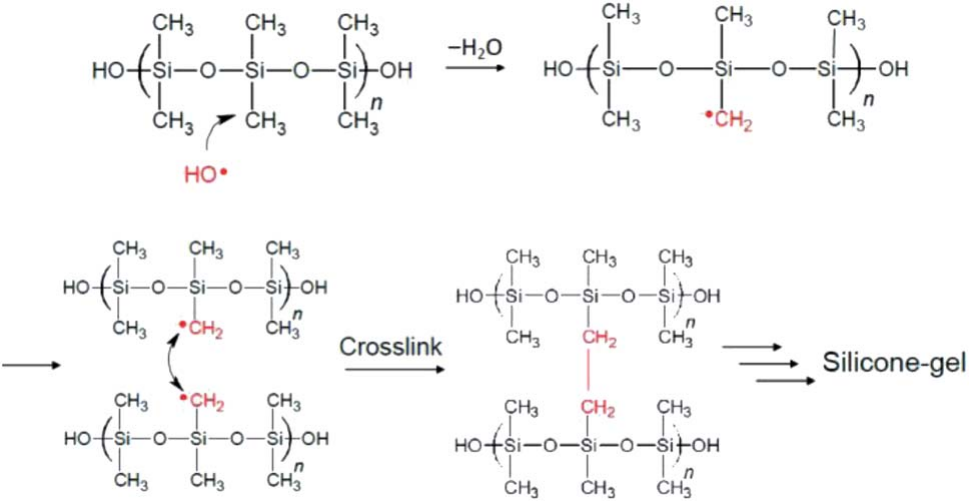
Inferred mechanistic stages of the synthesis of the silicone gel by the MILP method; only the stages of formation of the Si–˙CH_2_ radicals and the ultimate cross linking are shown. Not shown is the addition of ˙OH radicals onto other Si–˙CH_2_ radicals to yield Si–CH_2_OH pendant species that increases the relative quantity of O *vis-à-vis* other elements (see Table 3).

All available data reported herein allow us to infer a reaction scheme (Fig. 11) for the synthesis of the MILP silicone gel. Comparison with the conventional method, the silicone gel synthesized by the submerged plasma is cross-linked *via* C–C bond formation (Fig. 10a-ii and b-ii). We surmise that the initial reaction likely involved ˙OH radical attack of the methyl group in the silicone to yield macromolecular Si–˙CH_2_ radicals that cross-link to each other and ultimately, through various additional stages, lead to the gelation of silicone. As well, the high wettability of the MILP silicone gel and the large amount of oxygen (XPS results) suggest addition of OH groups to the silicone during the gelation (see also caption to Fig. 11).

**Fig. 12:**
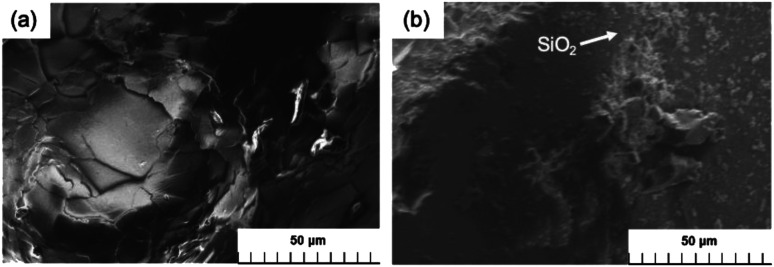
Comparison of SEM images of the silicone gel synthesized by (a) the MILP method and (b) by the conventional chemical synthesis method.

The conventional silicone gel and the MILP silicone gel were vacuum-dried, and then examined by scanning electron microscopy (SEM) and by viscosity measurements. The SEM photograph of the surface of the MILP silicone gel shows it to be smooth with no other solid matter (Fig. 12a), whereas the SEM image of the conventional silicone gel also reveals the presence of some silica (SiO_2_) particles formed from the hydrolytic transformation of the tetraethyl orthosilicate cross-linker (Fig. 12b).

Viscosity measurements carried out at an angular velocity of 10 rad s^−1^ yielded a viscosity of 0.0365 Pa s for the conventional silicone gel, while the viscosity of the MILP silicone gel was 2.28 Pa s (*i.e.*, 62 times greater). This is because gelation of silicone by the MILP method occurred through sufficient cross-linking with little unevenness of partial cross-linking. In contrast, gelation of silicone by the conventional method was accompanied by the hydrolysis of the TEOS orthosilicate that yielded silica particles, a part of which inhibited the cross-linking of silicone. In contrast, the viscosity of the MILP-formed PVP gel was significantly lower than the viscosity of the PVP gel from the conventional method. This is likely due to the water-solubility of the PVP polymer and thus easily attacked by ˙OH radicals. Moreover, the cross-linking and decomposition processes proceed competitively in the PVP gelation. As well, to the extent that the silicone gel is hydrophobic, the cross-linking likely occurs as a result of an uneven progress of the reaction with ˙OH radicals.

## 4. Concluding remarks

This study has shown that active radical species such as ˙OH radicals (among other players), generated during the formation of microwave-induced in-liquid-plasma (MILP), participate in the gelation process by attacking the macromolecules and cause a cross-linking reaction toward gelation of these macromolecules. Plasma density, cross-linking reaction time, and solution pH (among others) are important factors for efficient gel formation from macromolecules in aqueous media. One advantage of the MILP method in producing gels is that there is no need for toxic reagents such as cross-linking agents and initiators as they would be needed in the conventional chemical synthesis method. In addition, the study has demonstrated that not only the water-soluble polyvinyl pyrrolidone (PVP) polymer, but also the hydrophobic and water-insoluble silicone can be gelled. Unlike the conventional method, however, which requires a gelation time of several hours to days for the silicone, the MILP method requires but only about 5 min to cause silicone to gel in aqueous media, a clear advantage when considering industrial processes.

The *raison d'être* of the as-synthesized gels is in their several applications such as, for example, in environmental purification, in foodstuffs, and in the biological fields. For instance, addition of a biodegradable polymer to a wastewater contaminated with a radioactive substance (*e.g.*, cesium) that is subsequently followed by MILP irradiation can lead to the on-site removal of the radioactive substance *via* trapping the substance by the resulting gel.

## Conflicts of interest

There are no conflicts to declare.

## Supplementary Material

RA-011-D1RA03007H-s001

## References

[cit1] Goldston R. J. and Rutherford P. H., Introduction to plasma physics, Taylor & Francis, New York, 1995, ch. 1, pp. 1–17

[cit2] W. Crookes, “On radiant matter”, a lecture delivered to the British Association for the Advancement of Science, Sheffield, UK, August 22, 1879, accessed April 4, 2021, https://archive.org/details/OnRadiantMatter/mode/2up

[cit3] Clements J. S., Sato M., Davis R. H. (1987). Preliminary investigation of pre-breakdown phenomena and chemical reactions using a pulsed high-voltage discharge in water. IEEE Trans. Ind. Appl..

[cit4] Nomura S., Toyota H. (2003). Sonoplasma generated by a combination of ultrasonic waves and microwave irradiation. Appl. Phys..

[cit5] Horikoshi S., Serpone N. (2017). In-liquid plasma: a novel tool in the fabrication of nanomaterials and in the treatment of wastewaters. RSC Adv..

[cit6] Narushima T., Yoshioka T., Miyazaki H., Suga I., Sato S., Yonezawa T. (2012). Preparation of copper particles by microwave induced plasma in liquid process. J. Jpn. Inst. Met. Mater..

[cit7] Ishida Y., Motokane Y., Tokunaga T., Yonezawa T. (2015). Plasma induced tungsten doping of TiO_2_ particles for enhancement of photocatalysis under visible light. Phys. Chem. Chem. Phys..

[cit8] Shimizu Y., Nakamoto Y., Hyodo T., Egashira M. (2003). Decomposition of organic compounds by microwave-induced plasma in Liquid phase. Electrochem.

[cit9] Horikoshi S., Sawada S., Sato S., Serpone N. (2019). Microwave-driven in-liquid plasma in chemical and environmental applications. III. Examination of optimum microwave pulse conditions for prolongation of electrode lifetime, and application to dye-contaminated wastewater. Plasma Chem. Plasma Process..

[cit10] Horikoshi S., Sato S., Abe M., Serpone N. (2011). A novel liquid plasma AOP device integrating microwaves and ultrasounds and its evaluation in defuorinating perfuorooctanoic acid in aqueous media. Ultrason. Sonochem..

[cit11] Tsuchida A., Shimamura T., Sawada S., Sato S., Serpone N., Horikoshi S. (2018). In-liquid plasma. A stable light source for advanced oxidation processes in environmental remediation. Radiat. Phys. Chem..

[cit12] Horikoshi S., Sawada S., Tsuchida A., Serpone N. (2020). Enhanced degradation of organic pollutants with microwave-induced plasma-in-liquid (MPL): Case of flame retardant tetrabromobisphenol-A in alkaline aqueous media. J. Oleo Sci..

[cit13] Horikoshi S., Abe H., Torigoe K., Abe M., Serpone N. (2010). Access to small size distributions of nanoparticles by microwave-assisted synthesis. Formation of Ag nanoparticles in aqueous carboxy-methylcellulose solutions in batch and continuous-flow reactors. Nanoscale.

[cit14] United States Environmental Protection Agency, Basics of Green Chemistry, Green chemistry's 12 principles, Washington, DC 20460, accessed April 3, 2021, https://www.epa.gov/greenchemistry/basics-green-chemistry#twelve

[cit15] Kim G. D., Lee D. A., Moon J. W. (1999). Synthesis and applications of TEOS/PDMS hybrid material by the sol–gel process. Appl. Organomet. Chem..

[cit16] Wen J., Mark J. E. (1995). Sol–gel preparation of composites of poly (dimethylsiloxane) with SiO_2_ and SiO_2_/TiO_2_, and their mechanical properties. Poly. J..

[cit17] Nguyen N. T. B., Tu T. N., Bae W., Dang C. T. Q., Chung T., Nguyen H. X. (2012). Gelation time optimization for an HPAM/chromium acetate system: The successful key of conformance control technology. Energy Sources, Part A.

[cit18] Fukui K., Yonezawa T., Nagata C., Shingu H. (1954). Molecular orbital theory of orientation in aromatic, heteroaromatic, and other conjugated molecules. J. Chem. Phys..

[cit19] Kahn S. D., Pau C. F., Overman L. E., Hehre W. J. (1986). Modeling chemical reactivity. 1. Regioselectivity
of Diels-Alder cycloadditions of electron-rich dienes with electron-deficient dienophiles. J. Am. Chem. Soc..

[cit20] Fumagalli F., Kylián O., Amato L., Hanuš J., Rossi F. (2012). Low-pressure water vapour plasma treatment of surfaces for biomolecules decontamination. J. Phys. D: Appl. Phys..

[cit21] Kregar Z., Krstulovic N., Vukelić N. G., Milošević S. (2009). Space and time resolved optical emission spectroscopy characterization of inductively coupled RF water vapour plasma. J. Phys. D: Appl. Phys..

[cit22] Nomura S., Toyota H., Mukasa S., Takahashi Y., Maehara T., Kawashima A., Yamashita H. (2008). Discharge characteristics of microwave and high-frequency in-liquid-plasma in water. Appl. Phys. Express.

[cit23] Armstrong D. A., Huie R. E., Lymar S., Koppenol W. H., Merényi G., Neta P., Stanbury D. M., Steenken S., Wardman P. (2013). Standard electrode potentials involving radicals in aqueous solution: inorganic radicals. BioInorg. React. Mech..

[cit24] Rahma A., Munir M. M., Khairurrijal, Prasetyo A., Suendo V., Rachmawati H. (2016). Intermolecular interactions and the release pattern of electrospun curcumin-polyvinyl(pyrrolidone) fiber. Biol. Pharm. Bull..

[cit25] Okuyama M., Sato M., Akada M., Moriwaki T. (2010). Identification of excavated archaeological textile fibers and analysis of their degraded states by synchrotron radiation FT-IR microspectroscopy. Bunseki Kagaku.

[cit26] Mao H., Feng J., Ma X., Wu C., Zhao X. (2012). One-dimensional silver nanowires synthesized by self-seeding polyol process. J. Nanopart. Res..

[cit27] Fechine G. J. M., Barros J. A. G., Alcantra M. R., Catalani L. H. (2006). Fluorescence polarization and rheological studies of the poly(*N*-vinyl-2-pyrrolidone) hydrogels produced by UV radiation. Polym.

[cit28] Ogawa H., Ishikawa K., Inomata C., Fujimura S. (1996). Initial stage of native oxide growth on hydrogen terminated silicon (111) surfaces. J. Appl. Phys..

[cit29] Xu Y., Gao N., Gong Y., Huo S., Mohideen M. M., Hong S., Liu Y. (2019). Controllable preparation of methyltriethoxysilane xerogel nanofibers. J. Mater. Sci..

[cit30] Aghaei R., Eshaghi A. (2017). Optical and superhydrophilic properties of nanoporous silica-silica nanocomposite thin film. J. Alloys Compd..

[cit31] Zhong Y., Qiu X., Gao J., Guo Z. (2019). Chemical structure of Si–O in silica fume from ferrosilicon production and its reactivity in alkali dissolution. ISIJ Int..

[cit32] Gallis S. , Nikas V. and Kaloyeros A. E., Silicon Oxycarbide Thin films and nanostructures: Synthesis, properties and applications, Intech Open, 2017, vol. 14, pp. 277–302

[cit33] Bashouti M. Y., Paska Y., Puniredd S. R., Stelzner T., Christiansen S., Haick H. (2009). Silicon nanowires terminated with methyl functionalities exhibit stronger Si–C bonds than equivalent 2D surfaces. Phys. Chem. Chem. Phys..

[cit34] Han F., Yang S., Jing W., Jiang K., Jiang Z., Liu H., Li L. (2014). Surface plasmon enhanced photoluminescence of ZnO nanorods by capping reduced graphene oxide sheets. Opt. Express.

[cit35] Li X., Ding R., Shi W., Xu Q., Ying D., Huang Y., Liu E. (2018). Hierarchical porous Co(OH)F/Ni(OH)_2_: A new hybrid
for supercapacitors. Electrochim. Acta.

[cit36] dos Santos F. C., Harb S. V., Menu M. J., Turq V., Pulcinelli S. H., Santilli C. V., Hammer P. (2015). On the structure of high performance anticorrosive PMMA–siloxane–silica hybrid coatings. RSC Adv..

[cit37] Zhong Y., Qiu X., Gao J., Guo Z. (2019). Chemical structure of Si–O in silica fume from ferrosilicon production and its reactivity in alkali dissolution. ISIJ Int..

