# Supplementary material for: A novel green chemistry gelation method for polyvinyl pyrrolidone (PVP) and dimethylpolysiloxane (silicone): microwave-induced in-liquid-plasma
Source: RSC Adv. 2021 Jul 12;11(39):24326–35. doi: 10.1039/d1ra03007h (PMC9036679; doi:10.1039/d1ra03007h)
Supplement: RA-011-D1RA03007H-s001 [file RA-011-D1RA03007H-s001.pdf]

Electronic Supplementary Material (ESI) for RSC Advances.
This journal is © The Royal Society of Chemistry 2021

A Novel Green Chemistry Gelation Method for Polyvinyl Pyrrolidone (PVP) and Dimethylpolysiloxane (Silicone): Microwave-induced in-liquid-plasma

Satoshi Horikoshi,[a]* Seiya Sawada,[a] and Nick Serpone [b]

[a] *Department of Materials and Life Sciences, Faculty of Science and Technology, Sophia University, 7-1 Kioicho, Chiyodaku, Tokyo 102-8554, Japan*

[b] *PhotoGreen Laboratory, Dipartimento di Chimica, Università di Pavia, Via Taramelli 12, Pavia 27100, Italia.*

Supplementally Table and Figures

Table S1 Toxicity for LD_{50} (lethal dose 50) of initiators, crosslinkers and organic solvents [ref. 1]

	Major chemicals	LD_{50}(rat) / mg kg^{-1}
Crosslinker	N-methylenebisacrylamide	1390
	Ethylene glycol dimethacrylate	3300
Initiator	Azobisisobutyronitrile	100
	Benzoyl peroxide	7710

Ref. 1: M. Hirata, Latest water-soluble polymers, J. Jpn. Soc. Colour Mater., 1976, 49, 355-363.

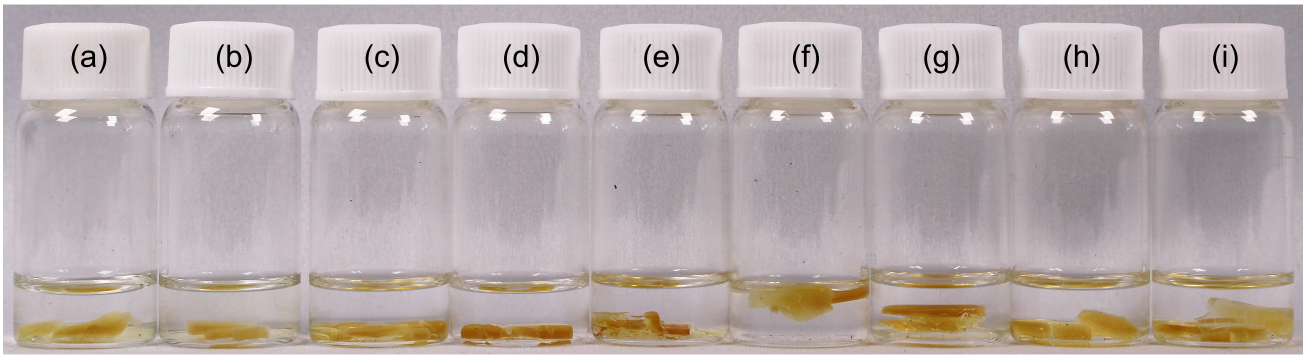

Figure S1. Photograph of in 9 kinds of solvents for a PVP gel generated by 5 min of microwave-induced plasma-in-liquid (MPL). Addition to ((a) water, (b) methanol, (c) ethanol, (d) acetone, (e) hexane, (f) chloroform, (g) dimethylformamide, (h) dimethylacetamide, and (i) tetrahydrofuran).

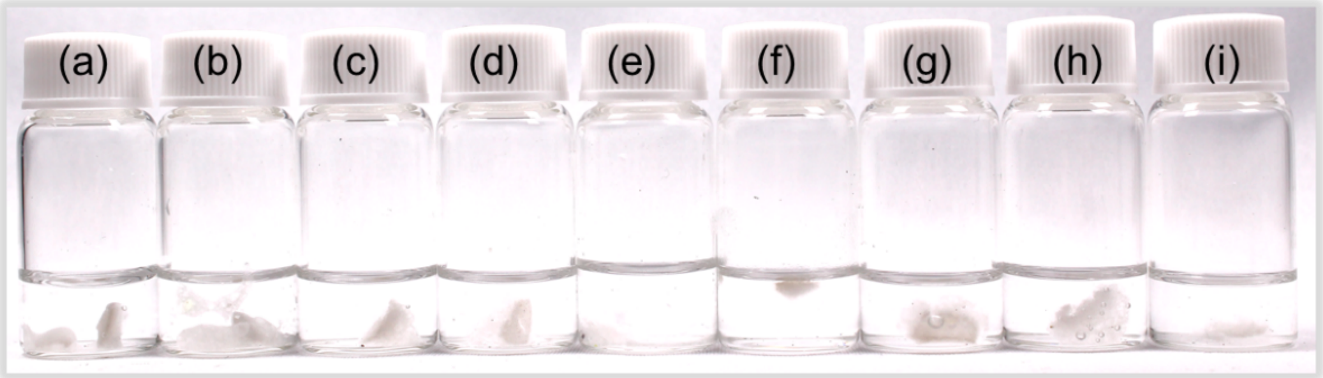

Figure S2. Photograph of in 9 kinds of solvents for a Silicone gel generated by 5 min of microwave-induced plasma-in-liquid (MPL). Addition to ((a) water, (b) methanol, (c) ethanol, (d) acetone, (e) hexane, (f) chloroform, (g) dimethylformamide, (h) dimethylacetamide, and (i) tetrahydrofuran).
